# The formation and function of the neutrophil phagosome

**DOI:** 10.1111/imr.13173

**Published:** 2022-11-28

**Authors:** Emily Naish, Alexander JT Wood, Andrew P Stewart, Matthew Routledge, Andrew Conway Morris, Edwin R Chilvers, Katharine M Lodge

**Affiliations:** ^1^ National Heart and Lung Institute Imperial College London London UK; ^2^ Medical School University of Western Australia Perth Australia; ^3^ Department of Critical Care University of Melbourne Melbourne Australia; ^4^ Department of Medicine University of Cambridge Cambridge UK; ^5^ Division of Immunology, Department of Pathology University of Cambridge Cambridge UK

**Keywords:** neutrophil, phagocytosis, phagosome

## Abstract

Neutrophils are the most abundant circulating leukocyte and are crucial to the initial innate immune response to infection. One of their key pathogen‐eliminating mechanisms is phagocytosis, the process of particle engulfment into a vacuole‐like structure called the phagosome. The antimicrobial activity of the phagocytic process results from a collaboration of multiple systems and mechanisms within this organelle, where a complex interplay of ion fluxes, pH, reactive oxygen species, and antimicrobial proteins creates a dynamic antimicrobial environment. This complexity, combined with the difficulties of studying neutrophils ex vivo*,* has led to gaps in our knowledge of how the neutrophil phagosome optimizes pathogen killing. In particular, controversy has arisen regarding the relative contribution and integration of nicotinamide adenine dinucleotide phosphate (NADPH) oxidase‐derived antimicrobial agents and granule‐delivered antimicrobial proteins. Clinical syndromes arising from dysfunction in these systems in humans allow useful insight into these mechanisms, but their redundancy and synergy add to the complexity. In this article, we review the current knowledge regarding the formation and function of the neutrophil phagosome, examine new insights into the phagosomal environment that have been permitted by technological advances in recent years, and discuss aspects of the phagocytic process that are still under debate.

## 1 INTRODUCTION

Neutrophils are among the first immune cells to respond following infection or injury; this makes the possession of proficient pathogen‐killing mechanisms essential. Phagocytosis, the process of detecting and engulfing particles into an organelle called the phagosome, is key to the ability of neutrophils to kill pathogens. In neutrophils, phagocytosis is a highly specialized and efficient event. Thus, neutrophils are the archetypal “professional” phagocyte, although monocytes, macrophages, eosinophils, and dendritic cells also display phagocytic ability to a somewhat lesser extent.^1^ As well as killing ingested pathogens, innate immune phagocytes may present antigens to adaptive immune cells, highlighting the importance of phagocytosis for both arms of the immune system.^2^ An intriguing neutrophil‐dendritic cell hybrid phenotype has been identified in mice, exhibiting retained phagocytic and microbial‐killing capacity as well as typical dendritic cell properties, such as antigen presentation.^3^

The neutrophil phagosome is a distinctive organelle, formed from an invagination of the plasma membrane to completely enclose an engulfed particle. A host of complementary processes and pathways then transforms the phagosome environment from a largely inert cellular inclusion into one optimized for the degradation of ingested particles.^4^ Within the phagosome, two major cytotoxic events take place: the production of nicotinamide adenine dinucleotide phosphate (NADPH) oxidase‐derived reactive oxygen species (ROS) and the delivery of microbicidal proteins from pre‐formed granules. The potential of these mechanisms to wreak havoc intracellularly and extracellularly necessitates exhaustive and complex regulatory systems, as excessive or aberrant neutrophil activation has been implicated in tissue damage in multiple inflammatory and autoimmune diseases.^5^

The complex mechanisms governing the phagocytic process in neutrophils have been a particular area of contention. Neutrophils are challenging to study, in part due to their short life span, abundance of degradative enzymes, and significant challenges in genetic manipulation.^6^ As a result, there is a relative paucity of neutrophil‐specific phagocytosis research. Many aspects of phagosome maturation have been studied in macrophages and then assumed to apply to neutrophils, despite disparities in phagocytic function (reviewed in [7]). One notable difference is that in most phagocytes (including macrophages), phagosome maturation follows an endocytic maturation pathway, whereby the phagosome fuses with lysosomes to form a phagolysosome,^7^ whereas in the neutrophil, the process of maturation is more rapid due to the presence of pre‐formed granules situated within the cytoplasm. This, combined with the neutrophil's streamlined killing mechanisms, leads to a very different phagosome environment. Despite these differences, study of the phagosome in other cell types has been informative to a degree as certain parallels can be drawn.

**FIGURE 1:**
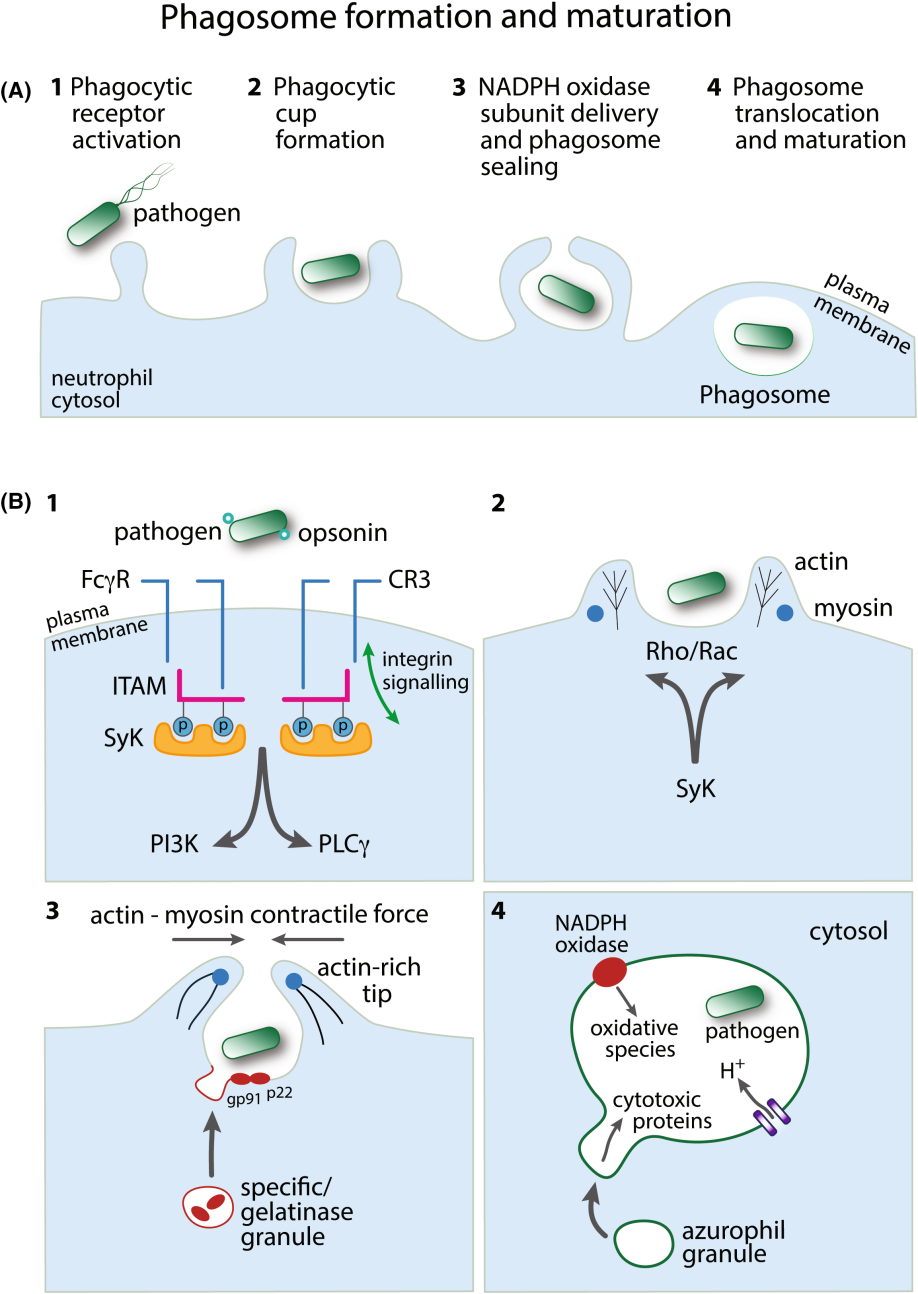
Phagosome formation and maturation. (A) Overview of the major steps in neutrophil phagosome formation following pathogen detection. Events 1–4 are depicted in further detail in panel B. (B) 1: An opsonized pathogen engages Fc receptors (FcγR) or complement receptors (e.g., CR3) to initiate phagocytosis. Both FcγR and CR3 can employ immunoreceptor signaling pathways: SH2‐domain‐bearing proteins (e.g., Syk) associate with phosphorylated ITAM, signaling downstream through phosphatidylinositol 3‐kinase (PI3K) and/or phospholipase Cγ (PLCγ). CR3 also employs independent inside‐out and outside‐in integrin signaling pathways. 2: Phagocytic receptor signaling induces regulation of the actin cytoskeleton via Rac and/or Rho. Myosin motor control of actin rearrangement drives extending pseudopod protrusions from the plasma membrane to form the phagocytic cup around the pathogen. 3: Cytosolic specific/gelatinase granules deliver proteins to the membrane of the forming phagosome, for example, the membrane‐bound subunits of NADPH oxidase, gp91^phox^ (NOX2), and p22^phox^. Actin polymerization at the pseudopod tips facilitates membrane sealing to complete the phagocytic vacuole around the pathogen. 4: The formed pathogen‐containing phagosome translocates toward the granule‐rich centriole within the neutrophil cytosol. NADPH oxidase generates antimicrobial reactive oxygen species inside the phagosome. The negative charge generated by this process is compensated by an influx of protons. Cytosolic azurophil granules, containing cytotoxic proteins, for example, elastase, fuse with the phagosome membrane to deliver their contents to the lumen of the phagosome

In this article, we will review the processes and mechanisms which shape the environment of the neutrophil phagosome and how these aid its pathogen‐killing abilities, including the formation of ROS by NADPH oxidase, the movement of ions across the phagosomal membrane, the pH of the formed phagosome, and the delivery of granule contents. We will highlight controversies in the complex field of neutrophil phagocytosis, where further investigation is paramount to understand their role in health and disease, which is often a fine balance between harm and help (Figure 1).

## 2 STUDYING THE PHAGOSOME THROUGHOUT HISTORY

Elie Metchnikoff (1845–1916), termed the father of cellular innate immunity, was the first to identify phagocytosis as a central host defense mechanism. He initially observed motile cells encircling an inserted thorn in starfish larvae, following which he realized the broader significance of phagocyte recruitment in host defense.^8^ At around the same time, Paul Ehrlich identified complementary humoral adaptive immunity. Ehrlich's precise staining techniques led to the modern era of leukocyte biology; his neutral dyes were able to identify “epsilon granules” in neutrophils, which he called “cells with polymorphous nuclei”.^8^ In 1960, it was shown that, following engulfment of bacteria, neutrophils underwent granule fusion (degranulation) with the phagocytic vacuole, and that the contents of the granules were “consumed,” indicating their usage against pathogens.^9^ A substance named “phagocytin” was the first microbicidal granule element to be identified,^10^ which was later shown to comprise a number of different cationic antimicrobial proteins.^11^ Subsequently, the advancement of staining and cell fractionation techniques, and cell imaging modalities including electron microscopy established the granule subset classification we know today, of which azurophil, specific, and gelatinase are the predominant subtypes.

Research into neutrophil phagocytosis has been full of contradictions and controversies. Shortcomings have been in part due to the inherent limitations of all in vitro and ex vivo studies. As neutrophils do not proliferate in vitro, much research has been undertaken using freshly isolated human donor cells, meaning donor‐dependent variation is unavoidable. The neutrophil‐like cells differentiated from HL‐60 and PLB‐985 cell lines have been used as an alternative; however, these cells are imperfect models for phagocytosis as intracellular killing is much less efficient, potentially due to the lack of specific granules in HL‐60 cells.^12^ The recent use of swimming zebrafish larvae, which are small, transparent, and only utilize innate immunity, in combination with high‐resolution live imaging has been revolutionary in following neutrophil responses to microorganisms in vivo and in real time.^14^ Additionally, intra‐vital two‐photon microscopy has enabled real‐time live imaging of neutrophils phagocytosing bacteria or viral prey in murine lymph nodes, with important implications for antigen presentation.^13^, ^14^

Other difficulties in phagocytosis research have arisen due to the transience of the phagosome, and an array of confounding factors present within this organelle; these include phagosomal membrane potential, osmotic strength, pH of the phagosome and cytosol, and the complex interplay of many different ion channels, enzymes, and chemical reactions.^15^ However, recent technological advances have increased our knowledge of neutrophil phagocytosis: In particular, the use of automated fluorescence microscopy to observe phagosome formation and maturation^16^, ^17^ and gene‐targeting technology in murine neutrophils^18^ have permitted more focused experiments.

Despite information from these new experimental techniques, several controversies remain, including the role of ROS in establishing the intra‐phagosomal environment. The increased oxygen consumption seen during phagocytosis in neutrophils was originally thought to be due to mitochondrial respiration, until the production of ROS within the phagosome was discovered. However, the extent to which ROS are directly antimicrobial is still actively debated. Another area of continued uncertainty is the intra‐phagosomal pH; due to the difficulties of measuring pH in such a small volume, purported phagosomal pH values have varied hugely over the years, ranging from acidification to neutral to alkalinization.

## 3 PHAGOCYTOSIS SIGNALING

During phagocytosis, multiple signaling cascades are activated, which result in the rearrangement of the actin cytoskeleton into a nascent phagosome (i.e., the stage of the phagosome immediately following initial sealing), after which phagosome translocation toward the center of the cell occurs, alongside phagosomal maturation and pathogen killing.^19^, ^20^, ^21^

The magnitude of the neutrophil phagocytic response to pathogens is substantial, which is unsurprising given that a primary function of neutrophils is pathogen destruction. Recent work from our group demonstrated that although there were minimal changes in protein expression between neutrophils exposed to *Staphylococcus aureus* bioparticles for 15 minutes compared to untreated cells, approximately one third of the phospho‐proteome was altered.^22^ These datasets are publicly available in the PRIDE database (https://www.ebi.ac.uk/pride/ reference PXD017092). This dramatic change in protein phosphorylation indicates that *S. aureus* encounter and phagocytosis result in a significant signaling stimulus for the neutrophil. Reactome database^23^ pathways enriched in our dataset of phagocytosing neutrophils include Rho GTPase signaling, neutrophil degranulation, membrane trafficking and nuclear membrane breakdown, vesicle‐mediated transport and phosphatidylinositol signaling, which are discussed in detail below. The use of unbiased techniques to understand dynamic signaling in neutrophils has been hampered by the release of degradative enzymes during cell lysis, though our group^22^ and others^24^, ^25^, ^26^ have demonstrated these techniques are becoming feasible with modern technologies. It is anticipated that further insights into complex signaling networks will be gained through the ongoing application of these methods.

Phagocytosis is initiated by the engagement of various receptors on the surface of neutrophils, often by endogenous opsonic ligands such as immunoglobulins or iC3b, a product of the complement system. Opsonins coating the pathogen are recognized by the neutrophil, stimulating phagocytosis, and they play an additional role by helping to overcome the repellent forces between the neutrophil and the negatively charged cell wall of many bacteria.^27^ Microbial pathogen‐associated molecular patterns (PAMPs) also bind to a variety of neutrophil receptors, such as dectin‐1.^28^, ^29^ Thus, neutrophils can internalize both opsonized and non‐opsonized particles. Here, we focus on opsonin‐mediated phagocytosis signaling pathways activated by Fc gamma receptors (FcyR) and complement receptors, which exhibit distinct mechanisms.

FcyRs bind immunoglobulin G (IgG)‐coated targets whereas complement receptors bind activated complement‐coated targets. Following receptor activation, pseudopods form from the plasma membrane to produce a cup‐shaped enclosure of the target particle that is enabled by the rearrangement of the actin cytoskeleton.^30^ Actin polymerizes at the leading edge and around the phagosomal cup, and this polymerization persists until the constriction and closure of the phagosome^31^: As the phagosome matures, the actin network disassembles to complete closure of the phagosome cup and allow the fusion of granules.^32^, ^33^

Originally, pseudopod extension to surround a micro‐organism prior to engulfment was thought to be exclusive to FcyR‐mediated phagocytosis as macrophage studies described “sinking” of complement‐opsonized targets.^34^, ^35^ More recent live‐cell imaging of phagocytosis in macrophages has contested this, with evidence of membrane ruffles and protrusions encapsulating targets in complement receptor 3 (CR3)‐mediated phagocytosis.^36^, ^37^, ^38^ However, slower “sinking” phagocytosis also occurs as an alternative mechanism requiring less involvement of membrane extensions. Moreover, in macrophages, the sinking phenomenon was shown to be CR3‐dependent but FcγR‐independent, whereas both CR3 and FcγR appear necessary for the formation and closure of FcγR‐mediated phagocytic cups.^39^ This is likely to be the case in neutrophils also, as neutrophils with CR3 mutations displayed decreased ability to ingest IgG‐opsonized targets.^40^

### 3.1 FcyR receptors

Neutrophils express FcγRI (CD64), FcγRIIA (CD32), and FcγRIIIB (CD16),^41^ where the predominant Fc receptor subtypes are FcγRIIA and FcγRIIIB.^42^ The class I and III receptors form multimeric complexes, while class II receptors exist as monomers with a unique phosphorylation motif and inhibitory action when engaged.^43^ As circulating unbound IgGs are ubiquitous, phagocytes must be able to distinguish between these and IgG‐associated with particles or immune complexes. Immunoglobulin receptors are therefore activated by clustering, mediated by the simultaneous engagement of multiple ligands, as opposed to ligand‐induced conformational change.^44^, ^45^ Clustering induces the activation and recruitment of Src family kinases, which results in the phosphorylation of tyrosine residues in the immunoreceptor tyrosine activation motif (ITAM) within the FcyR signaling subunit.

Phosphorylation of ITAM generates docking sites for proteins bearing SH2 domains, including the tyrosine kinase, Syk. Subsequent phosphorylation of Syk leads to the recruitment of various signaling proteins to the activated FcyR complex. Highlighting the importance of Syk in FcyR‐mediated phagocytosis, Jaumouillé et al found that in macrophages, Syk regulated FcyR responsiveness by increasing lateral receptor mobility and clustering through a reduction in actin polymerization.^46^ Although this mechanism was not demonstrated directly, neutrophils from Syk‐deficient mice displayed a similar reduced ability to ingest IgG‐opsonized particles.^47^, ^48^

Phosphorylation of Syk leads to the recruitment of adaptor proteins to the activated FcyR complex, leading to the activation of lipid‐modification enzymes, including phosphatidylinositol 3‐kinase (PI3K) and phospholipase Cγ (PLCy). PI3K is responsible for the accumulation of phosphatidylinositol 3,4,5‐trisphosphate (PtdIns(3,4,5)P3), derived from phosphatidylinositol 4,5‐bisphosphate (PtdIns(4,5)P_2_). PtdIns(4,5)P_2_ is also the substrate of PLCγ, and hydrolyzes into inositol1,4,5‐triphosphate (Ins(1,4,5)P3) and diacylglycerol (DAG), which also act as messengers in the phagocytic signaling cascade.^29^

Despite the actions of PI3K and PLCγ, levels of PtdIns(4,5)P_2_ increase in the early stages of macrophage phagocytosis, accumulating at the site of particle engagement and at the pseudopod tips. Levels abruptly decrease upon internalization, as actin disassembles to allow phagosome detachment, suggesting that accumulation of PtdIns(4,5)P2 is associated with initial actin recruitment, and its hydrolysis is associated with subsequent actin degradation and remodeling.^32^, ^33^ This is evidenced by the reduced actin disassembly during phagocytosis of IgG‐opsonized latex beads when PtdIns(4,5)P2 hydrolysis was inhibited in macrophages.^33^ The role of PtdIns(4,5)P2 in neutrophil phagosomes is less certain. Similar phosphoinositide dynamics in the phagosomal cup have been demonstrated between macrophages and the neutrophil‐like HL‐60 cell line, although this study did not differentiate between PtdIns(4,5)P2 and PtdIns(3,4,5)P3 accumulation.^49^ Conversely, a study of human and murine neutrophils showed that although PtdIns(4,5)P2 was abundant in the plasma membrane, unlike macrophages it decreased rapidly during phagosome formation and was undetectable after sealing.^21^

The synthesis of PtdIns(3)P on phagosomal membranes appears to be universal across phagocytosis,^50^ where similar dynamics have been demonstrated in neutrophils and macrophages. The generation of PtdIns(3)P by Class III PI3K in the phagosome membrane aids in the assembly of NADPH oxidase, making it essential for phagosome maturation. PtdIns(3)P binds the PX domain of p40^*phox*^ (a component of NADPH oxidase) with high affinity and selectivity^51^ and is critical for intracellular ROS production: Neutrophils from a patient carrying a mutation in the p40^*phox*^ PX domain (compromising PtdIns(3)P binding) had markedly decreased production of intracellular ROS during phagocytosis with subsequent impaired bacterial killing capacity.^52^

### 3.2 Complement receptors

Complement receptors are categorized into CR1 and CR2, formed by short consensus repeat (SCR) elements; CR3 and CR4, which belong to the β2 integrin family; and CRIg, which belongs to the immunoglobulin Ig‐superfamily.^53^ CR3 (also called CD11b/CD18 or Mac‐1) is the most efficient phagocytic complement receptor.^54^, ^55^ CR3 receptors are αMβ2 integrins that are activated by outside‐in (binding to extracellular ligands) and inside‐out (intracellular protein binding that changes integrin conformation and thus affinity state) signaling (reviewed in^56^, ^57^). With regard to phagocytosis, the complement fragment C3bi, a potent serum opsonin, is the most important ligand; however, CR3 is promiscuous and can bind a range of other ligands, including extracellular matrix proteins, surface receptors, blood coagulation proteins, and microbial surface molecules.

Patients with leukocyte adhesion deficiency (LAD), a condition caused by genetic mutations in β2 integrins (and thus CR3), experience severe recurrent bacterial infections.^58^ Neutrophils from patients with LAD type 1 are CR3‐deficient and defective in phagocytosis when stimulated in vitro but appear to have normal unstimulated IgG‐dependent phagocytosis.^40^ This suggests that there are CR3‐dependent and CR3‐independent mechanisms of phagocytosis and that the recurrent infections in LAD patients may be associated with failure to increase phagocytosis in response to inflammatory stimuli. However, neutrophils from these patients also display significant migration defects so it is difficult to tease out the relative contribution of this impairment, compared with phagocytosis defects, to infection susceptibility in vivo.

Inside‐out activation appears critical for CR3‐mediated phagocytosis. Inside‐out activation involves the transduction of an intracellular signal to the cytoplasmic and then the extracellular domain of an integrin, with signaling often initiated by G protein coupled receptor (GPCR) or Toll‐like receptor (TLR) activation, or cytokine stimulation.^53^ Once inside‐out signaling has facilitated conformational changes, from low to high affinity, outside‐in signaling occurs.^38^ This always involves linkage of integrins to the actin cytoskeleton but, depending on the effector response required, recruits and activates different adaptor proteins (reviewed in^59^).

As well as the distinct signaling pathways described above, CR3 also employs an immunoreceptor‐like signaling mechanism through phosphorylation of ITAMs on receptor‐associated transmembrane adaptors: Neutrophils lacking ITAM‐containing adaptor proteins are partially defective in integrin‐mediated phagocytosis.^60^ Similar to FcyR signaling, protein adaptors provide docking sites for the SH2 domains of Syk kinases which are then activated, followed by downstream signaling events. This study also found that Src family kinase phosphorylation of the adaptor proteins was essential for the association of the SH2 Syk domains and phagocytosis. These signaling events are important for rapid target ingestion: In macrophages, deletion of Syk or ITAM adaptors accentuated the inefficient and slow sinking method of phagocytosis.^39^ An alternative mechanism, where inefficient phagocytic cups were formed via the extension of membrane ruffles, was also inhibited by the deletion of Syk or ITAM adaptors, suggesting that Syk signaling induces ruffling, a phenomenon which can also be observed during FcyR‐mediated phagocytosis.^39^

The majority of early work on integrin signaling was performed in macrophages, initially describing Fc*γ*R‐ or CR3‐mediated phagocytosis as two discrete mechanisms.^61^ More recently, it has been suggested that CR3 and its downstream effectors are essential for phagocytosis of both opsonized and non‐opsonized targets.^62^

### 3.3 GTPase and cytoskeleton dynamics

Following protein and lipid kinase activity, signaling cascades induce actin polymerization and localized membrane remodeling. Rho family small GTPases play a central role in actin dynamic regulation and can switch between an active (GTP‐bound) and inactive (GDP‐bound) state. FcyR‐mediated phagocytosis is thought to involve predominantly Rac1, Rac2, and Cdc42, whereas complement‐mediated phagocytosis utilizes Rho. Rac and Cdc42 direct lamellipodial and filopodial membrane protrusions, respectively, whereas Rho induces the assembly of contractile actomyosin filaments.^63^, ^64^ However, in macrophages, RhoG involvement has also been identified in FcyR‐mediated phagocytosis and iC3b‐opsonized particle uptake is greatly reduced in Rac1‐ and Rac2‐deficient cells,^36^ suggesting some crossover of these pathways. It is likely that this is also true for neutrophils but has yet to be established.

In neutrophils, FcyR‐mediated phagocytosis was markedly impaired in Rac2‐ but not Rac1‐deficient cells,^65^ indicating a non‐redundant role for the Rac2 isoform. A rare inhibitory Rac2 mutation has been described in a patient who presented with recurrent and severe bacterial infection; however, phagocytic capacity was not assessed directly and Rac2 is also implicated in granule translocation (section 7.4) and NADPH oxidase function.^66^ Due to its polybasic domain, Rac can interact with anionic phospholipids. Thus, as well as actin modulation, Rac has an important role as a targeting signal to localize and correctly position proteins at the phagosomal membrane. Faure et al. showed that the transient recruitment of Rac2 and the NADPH oxidase component, p47^phox^, to the phagosomal cup membrane of neutrophil‐like PLB‐985 cells at the beginning of phagocytosis was phosphatidylserine‐dependent, and that inhibition of this recruitment resulted in reduced ROS production.^67^ In murine neutrophils, assembly and activation of NADPH oxidase following FcyR‐mediated phagocytosis was completely dependent on Rac2 whereas following complement‐mediated phagocytosis, there was redundancy between Rac1 and Rac2. Further differences were identified, where the oxidase rapidly (less than 6 seconds) accumulated on sealed phagosomes formed in response to complement‐opsonized prey, but during slower (more than 10 seconds) phagocytosis of IgG‐opsonized targets the oxidase could assemble at the base of a forming phagosome.^68^

In FcyR‐mediated phagocytosis, fluorescence resonance energy transfer (FRET) stoichiometry of macrophages revealed that Cdc42 was activated early and localized to actin in the extending pseudopod, Rac1 was active across the phagocytic cup and during closure, and Rac2 was active predominantly during contractile activities and closure of the phagosome.^31^ Similarly, in neutrophils, Rac1 was preferentially recruited to actin‐rich pseudopods due to the negative charge generated by phospholipids whereas Rac2 localized to the intermediately‐charged phagosomal membrane.^69^ However, neutrophil and macrophage GTPase dynamics are not identical as genetic deletion or pharmacological inhibition of murine or human neutrophil Cdc42 does not impair phagocytosis, although bacterial killing was compromised.^70^ These spatio‐temporal phagocytic control mechanisms for the different GTPases suggest that differential regulation of these enzymes coordinates the localized membrane remodeling.

Intriguingly, the use of pharmacological depolymerization agents showed that an intact actin cytoskeleton is required to target uptake and Rac2 translocation to the site of particle attachment in neutrophil‐like PLB‐985 cells, but disruption of the actin cytoskeleton once phagocytosis had been initiated does not prevent translocation of Rac2 toward the phagosome.^71^ Indeed, actin depolymerization following FcγR‐crosslinking was shown to enhance the oxidative burst,^65^ indicating that the timing of actin assembly and disassembly is important.

The formation of membrane protrusions and closure of the phagocytic cup are facilitated by myosin “motors,” which can control actin assembly, crosslinking and rearrangement, and are thus important regulators of membrane deformation, protein localization and phagosome translocation during phagocytosis (reviewed in detail in^72^). Myosin contractility is thought to be particularly important in FcyR‐mediated phagocytosis in neutrophils, where inhibitors of myosin ATPase prevented particle uptake,^73^ and mechanical models suggest there is a requirement for protrusive force.^30^

Rho and Rac activation are thought to be downstream events that follow the phosphorylation of the guanine exchange factor (GEF) Vav by Syk.^36^, ^74^ Neutrophils from Vav knockout mice are significantly deficient in FcyR‐ and integrin‐mediated phagocytosis.^65^, ^75^ Downstream of Rho and Rac, the Arp2/3 complex is responsible for actin polymerization. In murine neutrophils, Rac1 and Rac2 isoforms were shown to differentially regulate actin assembly using different pathways, where Rac2 mediated the majority of its effect on actin via the Arp2/3 complex.^76^

During CR3‐mediated phagocytosis, actin polymerization is propagated by two distinct mechanisms via RhoA. In one pathway, RhoA can activate Rho kinase, which phosphorylates and activates myosin II, leading to recruitment of Arp2/3; inhibition of Rho kinase or myosin II activity results in reduced Arp2/3 recruitment and actin cup assembly in murine macrophages.^77^ A non‐redundant role for Arp2/3 in neutrophils is less certain, however, as neutrophils isolated from a patient with a rarely described *ARPC1B* deficiency (lacking Arp2/3) did not demonstrate any defect in phagocytosis.^78^ In a separate pathway, RhoA can recruit the actin nucleator mDia1, which is recruited to the phagocytic cup via the microtubule‐associated protein CLIP‐170.^79^ Inhibition of mDia1 reduces actin polymerization and particle uptake.^80^ Highlighting the importance of RhoA signaling, the complement product, C5a (an anaphylatoxin implicated in sepsis pathogenesis), inhibits RhoA activation in a PI3Kδ‐dependent manner, which prevents actin polymerization and reduces neutrophil phagocytosis.^81^ Importantly, neutrophils from critically ill patients exhibit a similar phenotype, that is, reduced phagocytosis and a failure to activate RhoA or polymerise actin, which may contribute to the increased risk of infection in this patient group.

Various actin‐binding proteins have been shown to be important during neutrophil phagocytosis. The mammalian actin‐binding protein 1 (mAbp1), an adaptor protein phosphorylated by Syk, has been implicated in complement‐mediated phagocytosis.^82^ Syk was found to be necessary for the translocation of mAbp1 to the site of engulfment during phagocytosis and down‐regulation of mAbp1 led to a depletion of clustered β2 integrins in high‐affinity conformation. This high‐affinity conformation is essential to generate the tensile strength required for phagocytosis and the absence of mAbp1 led to severe defects in β2 integrin–mediated phagocytosis.^82^ Inhibition of the actin‐binding protein, coronin‐1, has also been found to arrest phagocytosis. Coronin‐1 is recruited to the phagosomal cup early, alongside actin. This suggestion that it may regulate actin is supported by the presence of coronin‐1 at the leading edge of migrating cells.^83^ Coronin‐1 co‐localizes with filamins, the most potent actin crosslinkers, at the phagocytic cup, where it appears to be involved in non‐opsonic phagocytosis in response to PAMPs.^84^ In macrophage phagosomes, phosphorylated Syk and paxillin colocalize and recruit vinculin, which facilitates target internalization by anchoring F‐actin. These exact phosphorylation sites have not been detected in neutrophils, but it is likely that a similar process occurs.^38^

Overall, distinct signaling mechanisms characterize FcγR and CR3‐initiated phagocytosis, which results in different, though overlapping, processes for particle uptake and phagosome formation. In vitro, it is often necessary to dissect the roles of various signaling molecules individually. These experiments have highlighted important redundant and non‐redundant roles for selected proteins, which have also been identified clinically through mutations, albeit rarely. In practice, however, there is likely substantial crossover and synergy between the immunoglobulin and complement pathways, which optimizes phagocytosis in vivo.

## 4 FORMATION OF THE PHAGOSOME

### 4.1 NADPH oxidase

The formation of NADPH oxidase gives rise to some of the key changes in the intra‐phagosomal environment, starting with ROS production. In most cell types, ROS are produced as a by‐product from a variety of processes, such as mitochondrial respiration,^85^ but in neutrophils, the majority of ROS are actively generated by NADPH oxidase (NOX enzyme complexes). NOX enzymes are “professional” generators of ROS, as reducing molecular oxygen to form ROS is their sole enzymatic function. In leukocytes, NOX2 is the main catalytic subunit, which is highly expressed in neutrophils.^86^ The enzyme NOX2 is dormant in the circulating quiescent neutrophil: Activation requires an initial priming step followed by full assembly of the NADPH oxidase complex from membrane‐bound and regulatory cytosolic subunits.^86^

At rest, the two membrane‐bound subunits, gp91^phox^ (which is the main redox center, NOX2) and p22^phox^, are embedded in specific granules. Upon phagocytosis, and before the phagocytic cup is even sealed, these subunits are delivered to the phagosomal membrane by granule exocytosis.^87^ Translocation of additional regulatory cytosolic subunits to the membrane‐bound gp91^phox^/p22^phox^ heterodimer to form the active NADPH oxidase complex is tightly controlled via the activation of a series of kinases, including protein kinase A (PKA), phosphoinositide 3‐kinase (PI3K), and mitogen‐activated protein kinases (MAPKs).^50^, ^88^ The small GTPase, Rac, is also important for NADPH oxidase activation. Guanine nucleotide exchange factors (GEFs) convert Rac‐GDP to the active Rac‐GTP, which translocates to the phagosomal membrane and there binds gp91^phox^ and p67^phox^.^87^, ^88^, ^89^ The obligate binding partner, p22phox, ensures stability of the heterodimer and accommodates docking of the regulatory subunits.^89^

NADPH oxidase activation is a dynamic process whereby a cascade of kinase signaling, calcium release, and small GTPase (predominantly Rac2) activation lead to subsequent NOX2 activation.^90^, ^91^ Activation of voltage‐gated proton channels, chloride channels, and subsequent calcium fluxes is also utilized to regulate the enzyme complex.^87^ The activation process is differentially regulated by various receptors, depending on the stimulus.^92^ Robust activation of NOX2 is seen following FcγR and integrin receptor ligation. Other receptors, such as formyl peptide receptors, which detect bacterial cell wall products, or GPCRs, can activate the enzyme complex directly but to a lesser extent. Neutrophil priming is an important control mechanism, which prevents inappropriate triggering of ROS generation. Here, an initial priming stimulus, for example, ligand binding to TLRs or TNF exposure, generates a pre‐activated neutrophil phenotype, which results in a substantially enhanced activation of the NADPH oxidase when the cell encounters a second stimulus.^93^ The range of upstream signaling pathways indicates that the threshold for NADPH oxidase activation is high, and that the ROS response is heterogenous, dictated by which signal is received. This complexity ensures tight regulation, with the aim of avoiding prolonged inflammation and oxidative damage.

### 4.2 The production of ROS in the phagosome

ROS are chemically reactive radical and non‐radical derivatives of oxygen, the former containing at least one unpaired electron. NOX2 (gp91^phox^) is the main redox center, transferring two electrons provided by cytosolic NADPH via internal heme moieties to the phagosome, whereupon oxygen is reduced to superoxide (O_2_ ·^−^). ROS react with many biomolecules, including DNA, proteins, lipids, and carbohydrates, to cause damage. The study of ROS has historically been focused on their capacity to cause cellular toxicity. In the 1930s, the rapid release of ROS was found to be associated with the formation of hydrogen peroxide (H_2_O_2_),^94^ which can generate potentially cytotoxic derivatives, such as the highly reactive hydroxyl radical. These findings were reinforced by the early discovery of bacterial ROS‐eliminating enzymes, such as superoxide dismutase.^95^

### 4.3 Reactive oxygen intermediates

The long‐held consensus has been that the ROS produced by NOX2, derived from superoxide anions, worked directly to kill bacteria,^88^, ^96^, ^97^ although this has now been contested (section 8). The initial question was which product was key to the microbial killing? The direct product of NOX2, superoxide anion (O_2_ ·^−^), is thought not to be highly bactericidal as it is weakly reactive and unable to migrate far from the site of production. However, a few of its derivatives are more reactive and able to diffuse through pathogen membranes.^88^, ^98^

O_2_ ·^−^ readily forms several reactive products, including hydrogen peroxide (H_2_O_2_). This either happens spontaneously, or through superoxide dismutase catalysis from two O_2_ ·^−^.^88^ H_2_O_2_ is converted to other forms of ROS by the myeloperoxidase‐hydrogen peroxide‐halide system: Myeloperoxidase (MPO) catalyzes the oxidation of different substrates by H_2_O_2_, with the most common substrates being halides. The products are hypohalous acids, including hypochlorous acid (HOCl). H_2_O_2_ also interacts with transition metals, such as iron, to produce hydroxyl radicals (OH.). However, iron is bound to lactoferrin within the phagosome and so is unlikely to contribute to bacterial killing.^98^ In addition to catalyzing H_2_O_2_ oxidation, MPO has the potential to act as a superoxide dismutase, contributing to H_2_O_2_ production. Furthermore, superoxide anion can react with redox intermediates of MPO that could impact its chlorination activity.^99^ Whichever reaction kinetics are favorable within the phagosome likely influence the degree of MPO‐dependent bacterial killing.

H_2_O_2_ can also interact with O_2_^·−^ or HOCl to generate singlet oxygen (^1^O_2_), an electronically excited state of molecular oxygen that is highly reactive but short‐lived.^100^ Some studies have suggested its presence in neutrophil phagosomes,^101^, ^102^ and there is some evidence acquired using ^1^O_2_ and antibodies to support the formation of ozone in neutrophils.^103^, ^104^, ^105^ However, the abundance and microbicidal effects of ^1^O_2_ remain ambiguous due to the imperfect methods of detection.^106^

An alternative reaction occurs with nitric oxide and superoxide anions, which generates various downstream reactive nitrogen species (RNS). Evidence of RNS‐mediated microbial killing in neutrophils is variable, with some studies showing little effect of RNS deficiency on neutrophil phagocytosis or killing ability,^107^ while others demonstrated increased infection susceptibility.^108^, ^109^ Discrepancies are likely explained by the varying susceptibility of different pathogens to RNS, which is thought to be a more important mechanism in tuberculosis and *Salmonella* species infection.

### 4.4 The role of ROS in the phagosome

The complex nature of ROS production illustrates the dynamic environment of the phagosome. Early findings by Klebanoff suggested that the bactericidal mechanism of neutrophils was mediated through MPO‐catalyzed iodination and chlorination.^110^, ^111^ He hypothesized that MPO, rather than having a direct bactericidal effect itself, might act indirectly in the phagosome to catalyze the production of substances with potent antibacterial properties. Initial investigations using iodine, which, when oxidized with H_2_O_2_, become the potent germicide iodide, demonstrated that MPO was only bactericidal when combined with iodide ions and H_2_O_2_. MPO‐dependent iodination was traced within close proximity of phagocytosed bacteria using electron‐microscopic autoradiographs on silver grains and demonstrated that the reaction did not happen in resting neutrophils, or in those treated with an MPO inhibitor.^110^ Similar results were obtained with chloride, which is converted into HOCl^111^; further studies have shown evidence of chlorination inside the phagosome and concluded that the amount of HOCl produced in the phagosome is sufficient to kill engulfed bacteria.^112^, ^113^, ^114^, ^115^ These findings elucidated roles for the respiratory burst, H_2_O_2_, and MPO in the neutrophil. However, these results have been contested, with subsequent experiments suggesting that ROS are not directly microbicidal; this aspect is further explored in section 8.

## 5 METABOLISM DURING PHAGOCYTOSIS

Phagocytosis is an active process: Protein phosphorylation and the generation of ROS require an energy source. Unlike monocytes, whose oxygen consumption, and thus phagocytic capacity, is substantially reduced by mitochondrial respiratory chain inhibitors, neutrophils derive most of their energy from glycolysis, even under aerobic conditions.^116^, ^117^ Early studies of metabolism in phagocytosing neutrophils have shown varied results: Some studies indicated increased glucose utilization and lactate production while others did not.^118^, ^119^, ^120^, ^121^ Variations in host species, phagocytic prey, or the availability of extracellular glucose may explain these experimental discrepancies.

The accepted dogma is that neutrophils rely on glucose‐fueled glycolysis to provide the energy for phagocytosis. Recently, transcriptomic analysis of neutrophils from patients with sepsis compared with healthy controls revealed upregulation of glycolytic gene expression, as well as a reduction in phagocytosis when healthy neutrophils were treated with glycolytic pathway inhibitors.^122^ However, in vivo the availability of glucose may be limited, for example in a purulent exudate. Detailed studies using genetically modified mice have shown that intracellular glucose and glycogen shuttling, and glycogen storage capacity are all important for neutrophils to maintain their ability to generate ROS and kill bacteria in situations where nutrients are lacking.^123^, ^124^

## 6 ION CHANNELS

The movement of NADPH oxidase‐generated electrons into the phagosome requires an ionic current to balance the negative charge produced, without which there would be huge depolarization of the membrane. The enzyme complex is inhibited from 0 mV to +190 mV, at which point electron translocation is abolished, highlighting the necessity of an outward cation or inward anion flux.^125^ If the neutrophil phagosome pH remains close to neutral, as some studies suggest,^112^, ^126^, ^127^ most of the compensated charge must be due to the movement of protons (H^+^), which are osmotically neutral^128^; only up to 5% of the charge could be compensated by any other ion.

In the 1980s, Henderson et al. found NADPH oxidase to be electrogenic and suggested that the negative charge was wholly compensated by H^+^ movement into the phagosome.^129^ There was speculation that the passage of H^+^ could be through gp91^*phox*^ itself^130^, ^131^; however, bioinformatic analysis of known voltage‐gated cation channels, followed by cloning of the H^+^ voltage‐gated channel 1 (Hv1) gene and examination of Hv1 knockout mouse neutrophils, confirmed the existence of a specific voltage‐gated H^+^ channel.^18^

Hv1, activated at depolarizing voltages, opens or closes a voltage‐gated H^+^ channel. Usually voltage‐gated ion channels comprise both voltage‐sensing and pore domains, but as Hv1 lacks the pore domain it is highly selective and tightly regulated by the transmembrane pH gradient, as well as voltage.^132^, ^133^ In neutrophils, Hv1 is highly expressed and delivered to the phagosome by granules with, but remaining independent of, NOX2.^134^, ^135^ Vacuolar‐type ATPase (V‐ATPase) is another H^+^ transport mechanism that actively pumps H^+^ into the phagosome using ATP hydrolysis.

The electrogenicity of NADPH oxidase and the consequent need for charge compensation was classically thought to be a nuisance. However, it is possible that charge compensation mechanisms serve a purpose for the cell^136^ H^+^ flux from the cytoplasm into the phagosome not only compensates the electrogenicity of the NADPH oxidase, but helps prevent osmotic disruptions, provides substrates for H_2_O_2_ and HOCl generation, and minimizes pH disturbances, both in the phagosome and cytoplasm. The H_2_O and HOCl formed from H^+^ and electrons are membrane permeable and thus have no osmotic effect. Strong acidification of the neutrophil cytoplasm is avoided through consumption of H^+^ during the production of ROS, and significant alkalinization of the phagosome is avoided through the dismutation of O_2_^·−^.

Surprisingly, deletion of Hv1 does not prevent ROS production completely, suggesting that other charge compensation mechanisms either exist in parallel or can compensate for lack of H^+^ transport.^24^ Chloride (Cl^−^) has been suggested as a compensatory ion^137^; however, it is estimated that approximately 90% of the oxygen consumed by NADPH oxidase is utilized for MPO‐catalyzed HOCl production and, given this requirement for Cl^−^ supply, it is unlikely that Cl^−^ movement out of the phagosome is responsible for all of the charge compensation.^138^ In stimulated neutrophils, the cytosolic level of Cl^−^ is lower than the estimated concentration of 70 mM in the phagosome, suggesting active transport mechanisms enable Cl^−^ accumulation in the phagosome.^139^

Two Cl^−^ channels (ClC), the cystic fibrosis transmembrane conductance regulator (CFTR) and ClC‐3, have been identified on neutrophil phagosomes.^140^, ^141^ Neutrophils from Clcn3(−/−) mice display reduced phagocytosis and ROS generation, demonstrating its importance in neutrophil phagocytic function.^141^ It has been suggested that ClC‐3 is a Cl^−^/H^+^ antiporter rather than an ion channel, meaning it could extrude H^+^ against the electrochemical gradient.^142^, ^143^ This appears counter‐productive to the accumulation of H^+^ in the phagosome; however, ClC‐3 may be partly responsible for H^+^ leak out of the phagosome (section 6).

CFTR is a cAMP‐activated chloride channel, which contributes approximately 50% of the total halide transport activity in neutrophils and is responsible for maintaining phagosomal HOCl levels.^144^ CFTR is defective in patients with cystic fibrosis (CF)^145^; neutrophils from CF patients with the typical ΔF508 homozygous mutation exhibit significantly reduced CFTR content in the phagosomal membrane^146^ and have impaired HOCl production.^140^ Although there are additional reasons why CF patients suffer recurrent infections, an impaired antimicrobial environment in the neutrophil phagosome due to defective Cl^−^ transport may be an important contributing factor, and killing of *Pseudomonas aeruginosa* (a common pathogen in CF) by neutrophils has been shown to be chloride‐dependent (reviewed in^147^) (Table 1).

**TABLE 1:** Genetic defects compromising phagocytic machinery and their clinical implications

Clinical condition	Genetic mutations	Clinical consequence	Neutrophil phagosome phenotype
Chronic granulomatous disease (CGD)	Genes encoding components of NADPH oxidase. Commonest affected subunit: gp91^phox^ – X‐linked mutations in *CYBB* gene. Disease severity correlates with NOX2 function.	Recurrent severe bacterial and fungal infections, commonly lung, skin, and liver with abscesses. Persistent inflammation with granulomata.	Absent/diminished production of all phagosomal ROS. Reduced bacterial killing, for example, *S. aureus*.^148^, ^149^, ^150^, ^151^
Myeloperoxidase (MPO) deficiency	*MPO* gene encoding myeloperoxidase. Commonest mutation: R569W.	Predominantly asymptomatic unless concurrent diabetes. Increased susceptibility to *Candida* species infection.	Increased phagosomal H_2_O_2_ due to i) absent/diminished MPO‐catalyzed production of HOCl and ii) prolonged activity of NADPH oxidase. Possible increased bioactivity of granule proteins, for example, elastase due to reduced HOCl‐dependent inactivation. Slower *S. aureus* killing markedly reduced *C. albicans* killing.^152^, ^153^, ^154^, ^155^
Cystic fibrosis (CF)	Cystic fibrosis transmembrane conductance regulator *CFTR* gene encoding CFTR chloride channel. Commonest mutation: ΔF508. Disease severity correlates with CFTR function.	Abnormally viscous mucus affecting multiple organs, for example, lungs and pancreas. Recurrent bacterial, fungal, and mycobacterial lung infections.	Defective targeting of mutant CFTR to phagosomes. Impaired chloride transport into phagosomes. Reduced phagosomal HOCl production. Reduced *P. aeruginosa* killing.^144^, ^146^, ^147^, ^156^, ^157^
Elastase deficiency	*ELANE* gene encoding neutrophil elastase. Missense mutations cause severe congenital neutropenia (SCN), probably due to mis‐trafficked or unfolded elastase. Whole *ELANE* deletion occurs with chromosome 19p terminal deletions.	Unclear clinical effects of elastase deficiency versus neutropenia. Recurrent severe bacterial infection in SCN but not described with *ELANE* deletion. G‐CSF recovery of neutrophil count in SCN does not prevent lethal infection.	Abnormal granule structure and additional antimicrobial peptide deficiency, for example, α‐defensins in SCN neutrophils, may also affect MPO activity. Reduced *Escherichia coli* and *C. albicans* killing in SCN neutrophils despite G‐CSF treatment. Reduced particle ingestion in some SCN neutrophils.^158^, ^159^, ^160^, ^161^

The K^+^/ Cl^−^ cotransporter KCC3 is also postulated to be present on the neutrophil phagosome: KCC3‐deficient murine neutrophils exhibited reduced NADPH oxidase activity due to disturbances in the recruitment and phosphorylation of its subunits.^162^ Furthermore, KCC activity was increased synchronously with neutrophil activation and was required for ROS generation, supporting the theory that KCC3 contributes to microbial killing and potentially to charge compensation.

Calcium ion (Ca^2+^) transport through the phagosomal membrane is more contentious; although it has been thought to regulate phagocytosis,^163^ the source of Ca^2+^ is still debated and the requirement of Ca^2+^ release for phagosomal oxidase activity remains controversial. This ion does, however, play an important role in regulating signaling and cytoskeletal dynamics during particle ingestion and granule fusion.

Ionic fluxes are dictated by dynamic interactions of channels, transporters, and pumps on the phagosome, which change as the phagosome matures, and have not been fully elucidated. Changes in pH and vacuolar volume provide clues as to the movement of ions in and out of the phagosome, but research into the pH of the neutrophil phagosome has posed challenges.

## 7 PH OF PHAGOSOME AFTER INGESTION

The idea of phagosomal acidification has been in existence for as long as the concept of phagocytosis itself: Metchnikoff originally hypothesized that acidification was the cause of death for ingested organisms.^164^ However, the phagosomal pH of neutrophils is inherently different from other phagocytes as it acidifies more weakly, at least at early timepoints.^126^, ^165^ Over the years, there have been many contradictions regarding neutrophil phagosomal pH. Investigators initially reported an acidification,^164^ but further studies suggested a biphasic pH change, with initial alkalinization followed by a modest acidification to pH ~6.5.^165^, ^166^ Subsequently, the intra‐phagosomal pH was suggested to remain neutral.^126^, ^127^

Proponents of the biphasic pH change proposed that initial alkalinization was due to the consumption of H^+^ by the formation of H_2_O_2_, and that this alkalinity was beneficial as it optimized bacterial killing by granule proteases.^165^ This theory was supported by the sustained acidification seen when NAHPH oxidase is inhibited. Similarly, neutrophils from patients with chronic granulomatous disease (CGD) (which lack a functional NOX2) undergo a rapid and extreme acidification during phagocytosis, which is associated with impaired bactericidal function.^165^ In contrast to healthy neutrophils, CGD neutrophils did not display the usual phagosome swelling, which was found to be independent of hydrolytic enzyme content and degranulation. It was suggested that normal phagocytic vacuoles enlarge due to an increase in the osmotically active products of bacterial digestion but that this does not occur in CGD neutrophils as the extreme acidification impairs digestion and thus swelling.

The second step in the proposed biphasic pH change is that of modest acidification, occurring once the respiratory burst subsides, and suggested to enhance activity of proteins with acidic pH optima, for example, hydrolases. The H^+^ pump, V‐ATPase, was hypothesized to be coupled to NADPH oxidase activity and to be responsible for the moderate acidification. However, in opposition of this theory, the rate of superoxide anion production was unrelated to V‐ATPase activity and inhibition of the pump did not provoke alkalization of oxidase‐active phagosomes.^126^

Potential limitations of the studies suggesting biphasic pH change^165^ include use of the pH sensor fluorescein, non‐sealed phagosomes, and dye leakage.^127^, ^166^ In addition, pH changes to optimize granule proteins may not be necessary; there are examples of prominent neutrophil enzymes that potentiate MPO‐mediated killing even when heat inactivated, for example, elastase and cathepsin G.^167^, ^168^ This could be because their cationic nature enables strong, disruptive electrostatic interactions with microbial surfaces, that consequently cause the microbe to be more susceptible to attack.

The current consensus is that neutrophil phagosomes acidify more weakly (certainly compared to macrophages) or remain neutral, due to robust NADPH oxidase activity.^169^ This would certainly make biological sense as NADPH oxidase functions optimally at neutral pH, so maintenance of this would allow maximal ROS production.^170^ Indeed, studies of single cells^126^ and direct measurement of intra‐phagosomal pH^126^, ^127^, ^166^ support this. More recently, genetic deletion of Hv1 has confirmed its role in sustaining NADPH oxidase activity (as a key charge compensatory channel), controlling both the homeostasis of ROS production and pH.^169^ H^+^ consumption, which should alkalinize the phagosome, is counteracted by a combination of active pumping of H^+^ into the phagosome by V‐ATPase (in of itself not enough to cause acidification as previously thought) and passive movement of H^+^ through Hv1.

Consistent with the theory of maintaining neutral pH, Jankowski et al. found that activity of NADPH oxidase itself hindered acidification through several complementary mechanisms: impairing V‐ATPase recruitment to the neutrophil phagosome, thus reducing H^+^ delivery; increasing phagosomal membrane permeability, therefore increasing passive H^+^ back‐flux into the cytosol; and increasing luminal H^+^ consumption.^127^ The mechanisms of the impaired V‐ATPase recruitment and increased H^+^ leak are uncertain. Theories for the former include V‐ATPase inhibition by ROS and oxidase‐induced depolarization of the phagosomal membrane, causing a depressed flux of Ca^2+^.^127^ In support of these findings, Chemaly et al. confirmed an inverse correlation of intra‐phagosomal ROS production and V‐ATPase accumulation on phagosomes.^170^

**FIGURE 2:**
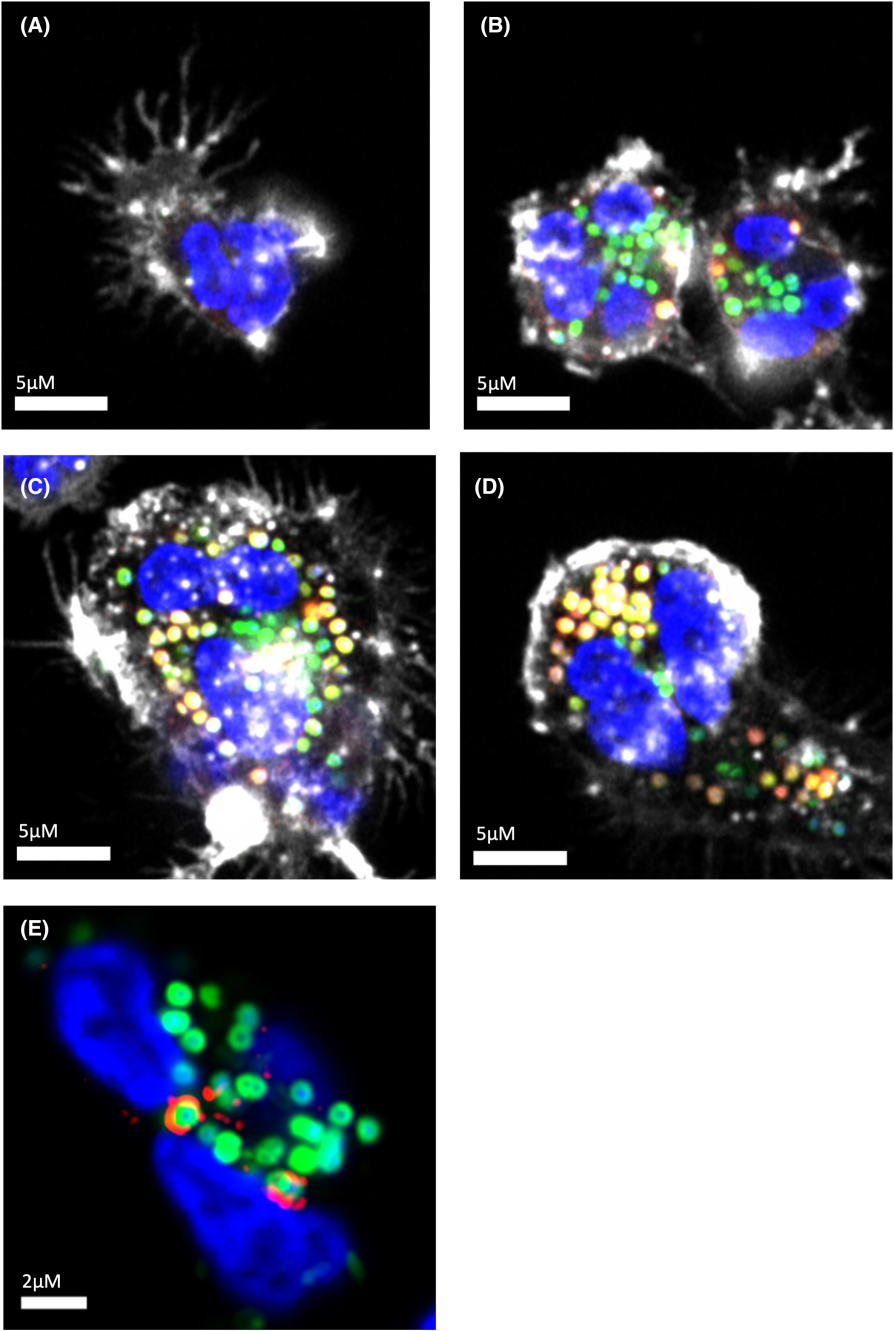
Maturation of phagosomes formed by human peripheral blood neutrophils ingesting Staphylococcus aureus. (A) Neutrophil that has not encountered bacteria (Blue: Nucleus labeled with Hoescht, White: Actin labeled with Alexa Fluor(AF)647‐phalloidin). (B) Early ingestion of S. aureus (Green: AF488‐labeled S. aureus bioparticles), with limited change in phagosomal pH as assessed by co‐localized lysotracker signal (Red: Lyso Tracker Red DND‐99, Yellow: co‐localization with AF488 S. aureus). (C): Mid‐maturation with mixture of low pH (mature) and high pH (immature) phagosomes. (D): Late maturation with the majority of phagosomes demonstrating low pH. (E) Early endosomal antigen 1 (EEA1) staining on the surface of S. aureus containing phagosomes. Note the penumbral rather than co‐localized signature indicating membrane distribution (Red: EEA1, mouse anti‐human EEA1 with secondary anti‐mouse AF568 phalloidin staining omitted for clarity)

Our group has generated recent evidence for the development of a more acidic pH as phagocytosis progresses (Figure 2). Using bacterial targets conjugated to a pH‐sensitive dye, we demonstrated progressive acidification of the maturing neutrophil phagosome at later time points (up to 120 minutes) that was class 3 PI3K‐ and V‐ATPase‐dependent.^22^ However, calibrated probes were not used at early time points to measure the exact pH. Of clinical relevance, exposure to the pro‐inflammatory complement product, C5a, reduced the proportion of neutrophils phagocytosing (as expected given its effects on RhoA signaling) but also impaired phagosomal acidification in neutrophils that had ingested prey, indicating a distinct mechanism of failed phagosomal acidification. The C5a‐mediated reduction in acidification was associated with a reduced phosphorylation of the V‐ATPase G subunit. Defective phagosomal acidification was also demonstrated in neutrophils from critically ill patients vulnerable to secondary infection, suggesting that acquired phagosome dysfunction in the context of systemic inflammation may increase susceptibility to infection.^24^ Another interesting observation is that when neutrophils phagocytose other apoptotic neutrophils, for example at sites of acute inflammation where neutrophils significantly outnumber macrophages, their oxidative burst is reduced with a resultant acidification of the phagosome, although pH was again measured at later timepoints (90 minutes).^171^ This demonstrates that the content of the phagosome may dictate its pH and consequently its maturation.

The disparity of phagosome pH in different phagocytes highlights the importance of pH regulation as a functional mechanism itself. The neutrophil's counter mechanisms against alkalinization and acidification suggest a likely neutral pH, certainly at earlier timepoints, with evidence of a later acidification as the phagosome matures. Further research is needed to examine the impact of systemic inflammation and extreme environments, such as the anaerobic hypoxic environment of infected or inflamed tissue, on pH regulation as the conditions may vary dramatically from in vitro studies. For example, the pH of the phagosome may be affected by the extracellular pH, due to knock‐on effects on the cytosolic and granule pH.

## 8 GRANULES

During the process of phagosome formation and maturation, granule fusion with the phagosome plays two fundamental roles: the delivery of antimicrobial granule proteins to the lumen and the insertion of functionally important membrane proteins into the phagosomal membrane, including receptors, ion channels, and gp91^phox^(NOX2)/p22^phox^. This fusion of pre‐formed granules is key to the neutrophil's rapid speed of engagement, engulfment, and killing of prey.^7^ Granules are estimated to make up about 40% of the phagosome volume^172^ and are commonly classified as peroxidase‐positive (azurophil) or peroxidase‐negative based on whether they contain MPO. However, they are heterogenous and can also be characterized by size, morphology, electron density, or protein content.^92^ The generally accepted subtypes are designated azurophil (primary), specific (secondary), and gelatinase (tertiary) granules, and secretory vesicles; this description is predominantly based on the timing of formation and subsequent differential contents, although there is a degree of overlap.^93^

Similar to signaling during phagosome formation, receptor ligation initiates intracellular signaling cascades during neutrophil degranulation (the process of granule delivery to plasma/phagosomal membranes). Although the signaling pathways that regulate degranulation are not completely delineated, activation of integrins,^173^ Fc receptors,^174^ and GPCRs^175^ has been implicated; the precise signaling pattern likely depends on the range, concentration, and context of external stimuli, and downstream signaling events may occur in parallel. As with activation of the NADPH oxidase, priming is also required for maximal degranulation response. However, these pathways converge on common degranulation effector mechanisms, principally cytoskeletal rearrangement, and granule/vesicle trafficking and membrane fusion.^176^

### 8.1 Granule contents

Neutrophil granules are produced from the Golgi complex by aggregation of immature transport vesicles; distinct stages of secretory activity produce granules with different contents (reviewed in^177^). However, they can also be classified by protein expression more loosely as a continuum. The prevailing hypothesis of granule heterogeneity is that of protein targeting by timing of biosynthesis, proposed by Borregaard et al. whereby concomitantly formed proteins are directed into granules together, and different granule subtypes are formed as protein expression changes during neutrophil maturation.^178^ However, it has been shown that active sorting of proteins to granules also occurs.^179^

Granules contain a range of different proteins: Enzymes and defense proteins are present in the lumen, whereas receptors, signaling proteins, and adhesion molecules exist in the membrane. Neutrophil granules mobilize to the plasma membrane for extracellular release and phagocytic receptor delivery (gelatinase and secretory vesicles) or to the phagosomal membrane for intracellular release (specific and azurophil).^180^ Specific granules are rich in antimicrobial substances, for example, lactoferrin and lipocalin‐2, and also contain membrane proteins, including NOX2.^10^ Azurophil granules are highly microbicidal, containing MPO, lysozyme, bactericidal/permeability‐increasing protein, defensins, and the serine proteinases elastase, cathepsin G, and proteinase 3.^7^ These proteins contribute to microbial killing in various ways, including inducing membrane permeabilization and lysis (e.g., cathelicidins and defensins) and scavenging metals needed for growth and enzyme function (e.g., lactoferrin).^181^, ^182^

### 8.2 Role of calcium in granule‐phagosome fusion

Sengeløv et al.^173^ showed an in vivo hierarchy of granule subtype mobilization, with complete mobilization of secretory vesicles, and a stepped reduction in the degranulation of gelatinase, specific and azurophil granules, respectively, that was dependent on intracellular calcium concentration.^175^ This hierarchical mobilization allows tight functional control, which is essential due to the large number of tissue‐destructive proteases contained within azurophil granules^183^; thus, their mobilization requires the most strongest of stimuli.

Granule fusion with the phagosome is inherently different from fusion with the plasma membrane due to the reversed curvature of the membrane^184^ and phagosome maturation. Defining the role of Ca^2+^ in phagocytosis has proven controversial; transient increases in cytosolic Ca^2+^ have been widely, but inconsistently observed during phagocytosis and the functional relevance and source of the Ca^2+^ remain a subject of debate (reviewed in^185^). Fusion of specific granules with phagosomes has been shown to be calcium‐dependent,^186^ but the role of calcium in azurophil granule fusion is uncertain.^187^ This distinction could be due to function and timing of fusion: Specific granules can fuse with the plasma membrane anywhere and deliver plasma membrane proteins to the phagosome, such as the subunits of NADPH, whereas azurophil granules mainly fuse with the phagosomal membrane and contain an array of enzymes and proteins that can damage surrounding tissue.^187^, ^188^ Nordenfelt et al. found that azurophil granule‐phagosome fusion and late‐stage killing in FcyR‐mediated phagocytosis was Ca^2+^‐independent but that a separate mechanism of early azurophil delivery in the forming phagosome was Ca^2+^‐dependent.^189^ This suggests that it is only a transient early phase of azurophil granule, and potentially other granule, fusion and killing that is Ca^2+^‐dependent and that later phagosome maturation mechanisms, such as ROS production and ion fluxes, make it redundant.

Actin polymerization is also independent of Ca^2+^ in neutrophils.^190^ Conversely, actin severing and depolymerization by gelsolin, which is crucial to dissolve the thick polymerized actin ring that surrounds the forming phagosome, is Ca^2+^‐dependent.^191^ Other possible targets for Ca^2+^ transients during phagosome formation include the Ca^2+^‐dependent protease calpain, which has been implicated in clustering of β2 integrins^192^; the Ca^2+^‐binding protein synaptotagmin, which translocates to CR3‐initiated phagosomes and is involved in particle uptake in macrophages^193^, ^194^; or the various Ca^2+^‐binding annexins, which promotes membrane fusion and may be involved in actin dynamics during phagocytosis.^195^, ^196^

### 8.3 Cytoskeletal reorganization

Actin polymerization, initiated by signaling cascades after receptor ligation, is essential for the formation of phagosomal cups: Inhibition of actin polymerization with cytochalasin B inhibits phagocytosis, although azurophil granule fusion with the phagosome persists.^197^ This suggests that the actin cytoskeleton may not be involved in granule‐phagosome fusion.

Microtubule polymerization is also promoted by phosphorylation signaling cascades in activated neutrophils (distinct from actin); late phagosomes are found in close proximity to the centriole, part of the microtubule organizing center (MTOC) where granules are rich.^198^ The use of colchicine, which binds to tubulin and prevents its polymerization into microtubules, led to phagosome disorganization and no preferential association with centrioles within the neutrophil, suggesting that microtubules are required to translocate the phagosome toward the granule‐rich centriole;^198^ in macrophages, this was observed directly.^199^ In neutrophils, colchicine also inhibited the association of MTOC and azurophil granules with the phagosome, implicating microtubule involvement in the movement of granules and their delivery to the phagosome, as well as movement of the phagosome itself.^197^ The association of kinesin, a motor protein involved in microtubule mediated transport, with neutrophil granules and microtubules also supports this theory.^200^

### 8.4 GTPases

The cytoskeleton's involvement in neutrophil trafficking is complex and the different granules are variously associated with actin and microtubules, which may explain how granules are differentially directed and regulated.^183^, ^197^ Actin and microtubule dynamics are predominantly controlled by Rac and Rab GTPases, which facilitate granule migration to the phagosome by orchestrating cytoskeletal rearrangement. Vesicle‐membrane tethering, docking, and fusion are then regulated by Munc family proteins and SNAREs (soluble NSF‐attachment protein receptors).

The precise secretory machinery associated with a particular granule is linked to its classification and function. Neutrophils display a non‐redundant role for Rac2 in the secretion of azurophil, but not specific or gelatinase granules: Neutrophils from mice deficient in Rac2 showed absent azurophil granule exocytosis whereas specific and gelatinase granule release was unaffected.^201^ Rac2 deficiency appears to mainly affect actin function in phagocytosis.^202^ Rac1 and Rac2 are also required for the formation of NADPH oxidase, depending on the micro‐organism phagocytosed.^203^

An example of directional granule trafficking control is demonstrated by Rab27a. Subcellular fractionation and immunoelectron microscopy has shown that Rab27a is located predominantly on gelatinase and specific granules, with lesser localization to azurophil granules.^204^ Rab27a directs all granules for plasma membrane fusion but granules lacking Rab27a are still able to fuse with the phagosome: Neutrophils from Rab27a‐deficient mice exhibited normal phagosome maturation and azurophil granule recruitment.^205^, ^206^ Conversely, Munc13‐4, an effector of Rab27a present on all three granule subtypes, was found to be essential for phagosomal maturation and delivery of azurophilic granules to the phagosome, with its absence leading to impaired intracellular bacterial killing.^205^ Therefore, Munc13‐4 is a protein of key significance in phagosomal killing due to the importance of MPO in formation of oxidized halides, and the delivery of toxic proteases and bactericidal peptides. Munc13‐4 may regulate granule tethering to the phagosome, which correlates with the finding that tethering of secretory lysosomes to the plasma membrane in cytotoxic T lymphocytes requires the Munc13‐4‐Rab27 complex.^207^ Alternatively, Munc13‐4 may form complexes with SNARE proteins to regulate azurophil granule fusion.

Regulation of differential granule fusion and mobilization is also mediated by Src tyrosine kinases and granule associated‐SNARE complexes, whereby association with certain family members dictates whether a granule is directed to the phagosome or plasma membrane.^208^, ^209^ For example, the Src family member, Hck, was found to be localized to azurophil granules, with translocation directed toward the phagosome^210^ whereas Fgr was associated with specific granules, and increased their fusion with the plasma membrane.^211^ Similarly, differential granule fusion is directed by various members of SNARE complexes.^208^, ^212^

Overall, a number of secretory control mechanisms ensure that the majority of azurophil granules (unlike specific and gelatinase granules) are preferentially targeted to the phagosome rather than the cell surface membrane, thus establishing a highly toxic phagosomal environment while limiting the capacity for host tissue damage.

## 9 OXIDATIVE VERSUS NON‐OXIDATIVE KILLING

Conventionally, the two arms of oxidative (ROS) and non‐oxidative (granule protein) killing in neutrophil phagosomes have been seen as opposing mechanisms, with evidence both for and against a directly microbicidal role for ROS in the phagosome. However, current research suggests a more synergistic approach.

As outlined in section 3, it was elucidated early on by Klebanoff that the respiratory burst in neutrophils produces a large amount of hydrogen peroxide (H_2_O_2_).^94^ As H_2_O_2_ is a potential substrate of MPO, the primary hypothesis was that MPO had an indirect microbicidal effect in the phagosome via MPO‐catalyzed oxidation of antimicrobial substances.^59^ In support of this theory, there is evidence of chlorination of ingested material,^112^, ^113^, ^114^, ^115^ and iodination is reduced in neutrophils from patients with MPO deficiency.^213^, ^214^ However, no loss of bacterial viability was observed in the pH range of 7.0–8.0, unless the H_2_O_2_ and iodide concentrations were markedly increased, suggesting that these results may not accurately reflect the phagosome environment. Similar findings were demonstrated using chloride but to a lesser extent.

Evidence for the importance of NOX2, and thus ROS generation, is provided by patients with CGD. CGD occurs as the result of a genetic mutation in any of the five subunits of NADPH oxidase and manifests as a primary immunodeficiency characterized by severe, prolonged, and often fatal infections. Neutrophils from CGD patients are unable to generate ROS, manifesting clinically as a profound susceptibility to bacterial and fungal infections. However, neutrophil microbial killing in these patients is not completely absent, which is best explained by the action of cytotoxic granule proteins (Table 1). This is demonstrated by the preserved ability of CGD neutrophils to kill *Escherichia coli*, where bacterial clearance is thought to be mediated by Bactericidal/permeability‐increasing protein,^215^, ^216^ indicating that non‐oxidative mechanisms can produce sufficient microbicidal effect against certain bacteria.

The original theory^217^ of a direct microbicidal role for ROS was subsequently contested (although the importance of MPO was not disputed). Segal et al. utilized stoichiometry to calculate that only a very small proportion of the oxygen consumed during the respiratory burst was utilized for iodination.^216^, ^217^ Furthermore, the pH used in previous experiments demonstrating chlorination, around 4.6–5.0, was thought to be too acidic, and it was also suggested that the initial in vitro experiments on the MPO/H_2_O_2_/Cl^−^ system had used unrealistically low granule protein concentrations. A later reassessment, using higher concentrations of superoxide radicals and granule proteins and a less acidic pH, reported that the directly microbicidal effects of H_2_O_2_ and HOCl were abolished.^218^ Segal's group proposed that as the majority of iodinated proteins were of host rather than bacterial origin, ROS may be ineffective against bacteria within the confines of the phagosome due to consumption of HOCl by the large amount of host‐derived protein. Despite this finding, there is evidence of HOCl‐mediated oxidation of bacterial methionine residues.^219^ Moreover, *E. coli* ingested by healthy neutrophils upregulated oxidant‐sensing genes (e.g., OxyR), and disruption of the bacterial methionine sulfoxide repair system (*ΔoxyRS* mutant) rendered *E. coli* more susceptible to neutrophil‐mediated killing.^220^ Intriguingly, neither upregulation of the oxidant‐sensing system or enhanced susceptibility of the mutant was observed when *E.coli* were phagocytosed by neutrophils from CGD patients.

An opposing theory of non‐oxidative killing was thus proposed whereby the microbicidal capacity of neutrophils was due to the abundant granule proteins instead of ROS. It was suggested that the purpose of the electrogenic current of NADPH oxidase was to drive compensatory ion fluxes to optimize the conditions in the phagosome for granule enzymes (rather than primarily to generate ROS), which could also explain why pathogen killing is impaired in CGD.^136^ However, whether any cations other than H^+^ significantly influx into the phagosome is debatable and depends largely on the pH of the phagosome: If the pH remains close to neutral (as currently thought), then the influx will be predominantly H^+^ whereas if the pH rises (initial alkalinization) then other cations must be involved (section 6). Calculations based on the small amount of osmotic swelling of the phagosome estimate that most of the charge compensation must be osmotically neutral.^128^ Thus, it is likely that the only cation entering the phagosome is H^+^.

Evidence for the importance of neutrophil granule enzyme‐mediated killing is provided by mice deficient in the proteases, cathepsin G, and elastase. Neutrophils from these mice exhibited normal respiratory burst, ROS production, and iodination, but the mice were unable to resist infection with *S. aureus* (a prominent cause of infection in CGD) or *Candida albicans* (which also causes severe infection in MPO‐deficient mice).^136^ Reeves et al. suggested a role for MPO in protecting granule enzymes against oxidative damage, for example cathepsin G, which is very sensitive to oxidation by H_2_O_2_. In this scenario, the impaired killing observed in neutrophils from MPO‐deficient mice^221^ was suggested to be due to increased accumulation of H_2_O_2_,^222^ potentially leading to the oxidative inactivation of antimicrobial proteins. Conversely, as HOCl can also inactivate antimicrobial proteins, such as elastase, a lack of MPO may actually enhance their activity. Assessing the role of single proteases in humans is even more complex; for example, mutations in elastase may cause disease through protein misfolding and mis‐trafficking, resulting in neutropenia, as well as from deficiency of the protease (Table 1).

The disparity in the clinical manifestations of CGD, where patients suffer significant infection‐related morbidity and mortality, and MPO deficiency, which somewhat surprisingly goes largely unnoticed, questions the predominance of a single killing mechanism. CGD patients experience severity of disease correlated to the degree of impairment of NADPH oxidase; however, the precise mechanism of antimicrobial defense due to NADPH oxidase is ambiguous.^148^ Although MPO deficiency in humans does not commonly present with critical illness,^223^ MPO‐deficient neutrophils exhibit less efficient bacterial killing^224^ (Table 1). The reduced killing speed observed in MPO‐deficient neutrophils *in vitro* ^152^, ^153^ suggests that MPO may be involved early, and that proteases, potentially acting later as the pH is optimized, can compensate for the deficiency in vivo. An important early report highlighted the potential compensatory contributions of peroxidase‐dependent and peroxidase‐independent microbicidal systems by comparing neutrophils from patients with CGD or MPO deficiency.^225^ Furthermore, when both systems are operational in healthy neutrophils, oxidative and non‐oxidative microbial killing may be synergistic, as demonstrated by the ability of elastase to potentiate MPO‐dependent killing of *S. aureus* or *E. coli* in vitro in an acidic environment.^167^

**FIGURE 3:**
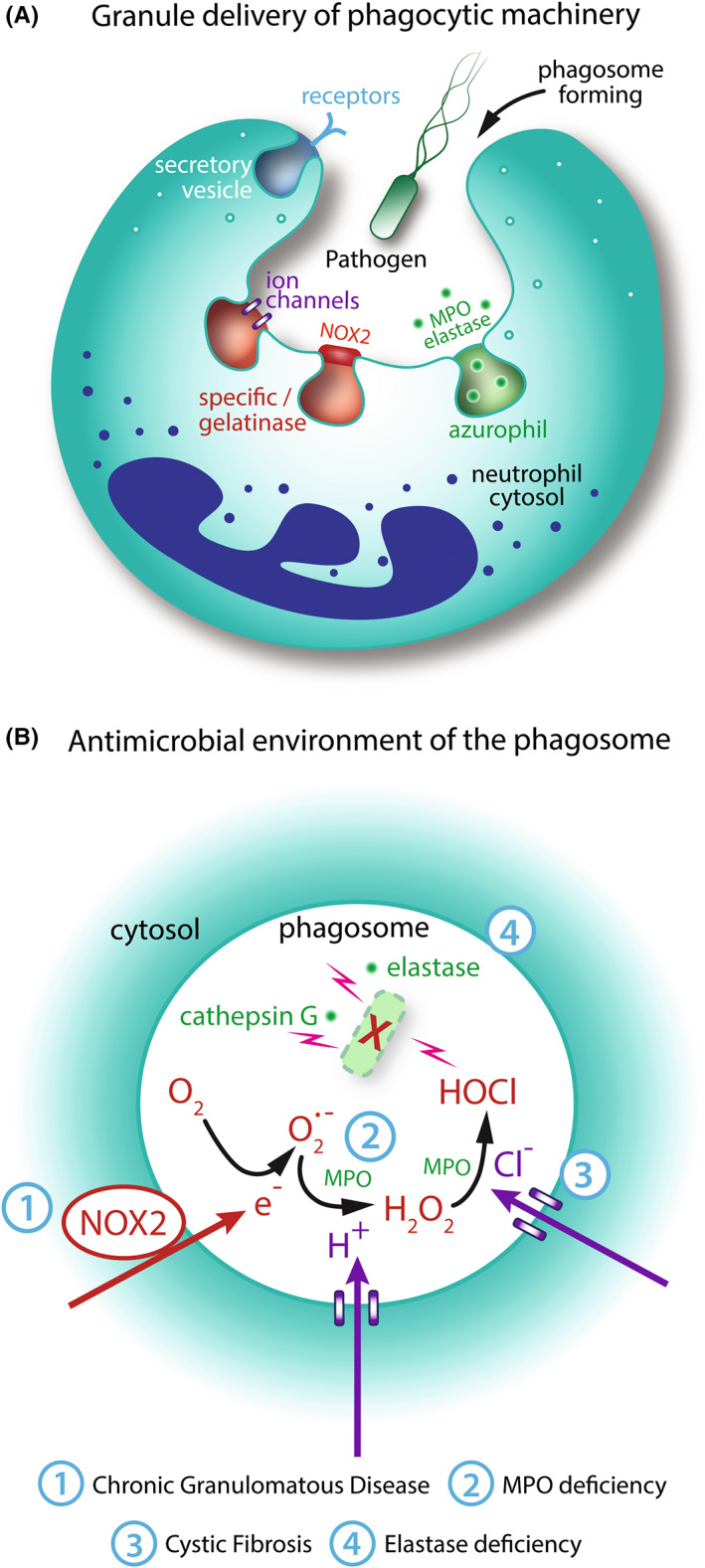
Role of granules and reactive oxygen species in phagosome formation and function. (A): Granules deliver phagocytic machinery to the plasma and phagosome membranes during phagocytosis. Secretory vesicles supply phagocytic receptors to the plasma membrane. Specific and gelatinase granules deliver NADPH oxidase components and ion channels to the phagosome membrane. Azurophil granules supply myeloperoxidase (MPO) and cytotoxic proteins and proteases, including elastase. (B) Reactive oxygen species (ROS) and granules contribute synergistically to microbial killing. NADPH oxidase translocates electrons into the phagosome which react with molecular oxygen to form superoxide (O2^.−^). Electrogenic charge is compensated by proton (H^+^) influx, predominantly through Hv1 channels. MPO catalyzes the reaction of O2^.−^ and H^+^ to form hydrogen peroxide (H_2_O_2_). Chloride (Cl^−^) enters the phagosome, for example, through CFTR channels. MPO oxidizes Cl^−^ to form HOCl which is likely to be directly microbicidal. Non‐oxidative proteins and proteases, for example, elastase and cathepsin G, are also directly microbicidal. Numbers in blue indicate clinical syndromes associated with mutations in various steps of the pathway (Table 1)

The mechanism of oxidative killing is still controversial. Evolutionarily, the theory that NADPH oxidase only produces ROS for the purpose of membrane potential changes is unlikely due to the toxicity of its products. However, as the lifespan of neutrophils is so short, it is questionable whether neutrophils need to be protected against ROS. Presently, the conclusion is that there is not a single predominant mechanism, but a complex synergistic relationship between oxidative and non‐oxidative killing that provides alternative compensatory systems, as is often the case with the immune system (Figure 3).

## 10 WHEN THE PHAGOSOME CANNOT FORM

Neutrophils are powerful and efficient killers, but bacteria are formidable opponents and with their rapid evolutionary speed they have developed a range of evasion tactics. A common evasion tactic of bacteria is the formation of aggregates that circumvent phagocytosis. Large fungal hyphae also obviate phagocytosis. However, ligation of the phagocytic dectin‐1 receptor, which detects fungal elements, can divert degradative proteins from the phagosome to the nucleus, thereby enabling the formation of neutrophil extracellular traps (NETs) in situations where a pathogen is too large to engulf.^226^ NETs, composed of decondensed chromatin acting as a scaffold for cytosolic and granule proteins, act to trap pathogens which cannot be phagocytosed, and can exert antimicrobial effects via exposed antimicrobial molecules.^227^

Metzler et al. found that in a subset of resting neutrophils there is a protein complex, termed the azurosome, which is localized on the azurophil membrane.^228^ This complex acts as a scaffold for proteins, including elastase and MPO, and is regulated by oxidation, specifically by H_2_O_2_. When activated by H_2_O_2_, some proteins, including elastase, dissociate from intact granules into the cytosol by an unknown mechanism. Elastase is then activated in an MPO‐dependent manner and is free to enter the nucleus and cleave histones, producing NETs.^228^ Thus, similar to pathogen killing during phagocytosis, NET‐generation may be dependent on both ROS and granule proteins.

The ability of neutrophils to “sense” pathogen size during attempted phagocytosis is intriguing. When neutrophils were exposed to *Candida albicans* hyphae, over 70% of total elastase was seen in the nucleus, accompanied by NET release, compared to less than 20% when *Candida albicans* was in the easily phagocytosable yeast form.^226^ The exact mechanism is unclear, but it is possible that the “decision” for NETosis versus phagocytosis is dependent on whether elastase is delivered to the phagosome: phagocytosis, which acts more rapidly than NETosis (seconds to minutes versus minutes to hours), sequesters elastase to the phagosome and inhibits the production of NETs. An additional response to fungal hyphae is mediated via CR3 ligation by the fungal PAMP, β glucan, and components of the extracellular matrix. Here, temporally regulated CR3 cross‐talk with different β_1_ integrins can instigate neutrophil swarming, whereby neutrophil aggregates can form a so‐called attack complex, prior to NETosis.^229^ This size‐dependent regulation ensures that the destructive collateral damage from NETosis is only permitted when phagocytosis is not a viable option. Intriguingly, NETosis does not appear to preclude the ability of neutrophils to also perform phagocytosis, and anuclear neutrophils containing bacteria have been observed, implying that NET generation and phagosome maturation can be compartmentalized within the same cell.^230^

This “decision making” during neutrophil phagocytosis highlights the complexity and plasticity of the neutrophil and shows that even when neutrophils are unable to form a phagosome, there are other microbicidal mechanisms they can employ to protect the host.

## 11 PHAGOSOME RESOLUTION

Phagosome resolution is the concluding stage in the phagocytic process, comprising disposal or recycling of ingested contents and membrane resources, as well as providing the opportunity for antigen presentation.^231^, ^232^ Resolution is an important step that allows phagocytes to return to homeostasis but, in comparison with phagosome formation and maturation, resolution remains largely unexplored. Consequently, the molecular basis of phagosome resolution is rather speculative, predominantly based upon related processes such as lysosome turnover and autophagy. Limited experiments in phagosomes have almost exclusively been performed using macrophages (reviewed in [4]), whose phagosomes mature through fusion with endosomes and lysosomes in a manner which is very different from neutrophil phagosome‐granule fusion. Therefore, although knowledge acquired from these experiments is often extrapolated, findings in macrophages during this phagocytic stage may not be truly representative of the situation in neutrophils.

The objectives of phagosome resolution are manifold: safe disposal of destroyed prey; waste management (including recycling) of ingested contents, including nucleic acids, proteins, and lipids; antigen presentation to lymphoid cells; and resorption of the phagosomal membrane. Resolution was originally thought to proceed in a similar fashion to unicellular eukaryotes, which egest indigestible content and plasma membrane.^233^ However, recent work in macrophages has shown evidence of shrinkage and fission,^234^, ^235^ with resolution of phagolysosomes through fragmentation, by vesicle budding, tubulation, and constriction.^236^ It is unclear how this reformation of lysosome‐like organelles during fragmentation would apply to neutrophils as their granules are pre‐formed. In addition to mechanical contraction, exportation of organic osmolytes produced by target degradation is essential for volume loss during phagosome resolution to prevent osmotically‐induced hydrostatic pressure (reviewed in^237^).

One conundrum of the phagosome maturation and resolution process is that phagocytes must be able to target the intra‐phagosomal lipids of ingested prey for degradation while maintaining an intact phagosomal membrane that is protected from lipolytic enzymes, but also be able to resorb this membrane once internal degradation is completed. The mechanisms controlling phagosomal membrane recycling are largely unknown but possibilities for the handling of various lipid species are discussed in.^4^

There is a particular paucity of information regarding signaling events for phagosome resolution. In macrophages, the fragmentation process is thought to involve phosphoinositides, particularly PtdIns4P, and Rab GTPases,^237^ which direct tethering to the endoplasmic reticulum.^235^ Whether this process occurs in neutrophils is uncertain.

Given that phagosome resolution is a pivotal step in the successful completion of phagocytosis, this stage has garnered surprisingly little attention. In neutrophils, the processes required for phagosome resolution are almost completely unknown. Macrophages must be equipped to perform multiple rounds of phagocytosis while maintaining phagocytic and degradative capacity but it is not clear how vital this step is in the relatively short‐lived neutrophil, which is itself efferocytosed by macrophages during inflammation resolution. Given the unique nature of neutrophil granules, and the involvement of proteins required for phagosome fragmentation throughout phagosome maturation, there will be considerable challenges in elucidating this process.

## 12 CONCLUSION

For the past few decades, progress in neutrophil biology has been extremely slow; many researchers have opted to study cell types that are more easily cultured and genetically manipulated. Over time, however, the neutrophil has been revealed as unique, and its plasticity and complexity underestimated. The view that neutrophils are single function suicide killers has been overtaken by evidence of integration of complex signals to make “decisions” and instigate a range of different signaling pathways in the face of invading pathogens. This is particularly apparent in the neutrophil phagosome, which has phenotypic and functional plasticity. We now know that the neutrophil phagosome environment changes to optimize killing, but an interesting debate still surrounds the complex interplay between various systems.

One key area of contention regards the antimicrobial role of ROS, which are small, transient, and ubiquitous, juxtaposed with that of granule proteins, which are macromolecules with specific killing mechanisms. For years, researchers have believed a single mechanism to be predominant; however, more recent research suggests synergism. This is highlighted by the different but overlapping consequences of CGD, MPO deficiency, and cathepsin G and/or elastase knockout. As may be expected, the ability of the compromised host to prevent bacterial or fungal infection also varies depending on the target organism.^136^, ^224^, ^238^, ^239^ It is likely that some pathogens are more susceptible to a particular mode of killing. For example, neutrophil killing of *S. aureus* appears to require ROS as cells experiencing a lack of molecular oxygen under hypoxia displayed impaired bacterial killing (but not ingestion).^240^ This makes sense in the context of the wider immune system, which employs a combination of different effector systems to tackle infection.

Another area of phagosome research which has not yet provided a conclusive answer is that of intra‐phagosomal pH. Variation in experimental techniques and theories has led to a range of different persuading arguments; however, there is consensus that neutrophils are unique phagocytes, with a weaker acidification than macrophages due to their increased ROS production, and it is likely that the phagosome acidifies more as it matures. The lack of decisive data on pH has also led to ambiguity about the phagosomal ion fluxes. H^+^ and some Cl^−^ transportation mechanisms are well established, but the involvement of other ions, such as K^+^ and Ca^2+^, is still unclear. Ion transport mechanisms are also intrinsic to other systems, such as MPO‐mediated chlorination and NADPH oxidase activity. This interconnected web of components and factors, which in turn react together to create many derivatives, are the crux of the dynamic phagosome. Consequently, trying to model the changing biochemistry of the phagosome in vitro is challenging and extrapolating from studies of the systems in isolation, for example, inherited defects and murine knockouts, can undermine the complexity of neutrophil phagocytosis.

Some aspects of the neutrophil phagosome are well established, for example, the formation of NADPH oxidase, production of ROS, and granule fusion mechanisms. However, due to the functional plasticity of neutrophil phagocytosis, we must continue to investigate the unique and distinct mechanisms to understand the complex interaction of neutrophils with each other, with various pathogens, and with other cells. Advances in proteomics, live imaging techniques, and genome sequencing have advanced our understanding in this fascinating fusion of cell biology and microbiology. To further our knowledge of neutrophil phagocytosis, we must employ careful experimental design and analyze potentially limited technological approaches, while acknowledging and appreciating the complexity of the immune system.

## CONFLICT OF INTEREST

Andrew Conway Morris is a member of the scientific advisory board of Cambridge Infection Diagnostics Ltd and reports speaking fees from Boston Scientific.

## Data Availability

The data that support the findings of this study are openly available in PRIDE PRoteomics IDentifications Database at https://www.ebi.ac.uk/pride/, reference number PXD017092.^241^
